# Differential Gene Expression Across Species Following Spinal Cord Injury: A Systematic Review and Meta-Analysis

**DOI:** 10.1007/s12035-025-05496-y

**Published:** 2025-12-20

**Authors:** Sara S. AbouZekry, Manar S. Abdullah, Ahmed S. Abouhashem, Sungsoo Chun, Ahmed Abdellatif

**Affiliations:** 1 Biotechnology Program, American University in Cairo, New Cairo, 11835 Egypt; 2 Helmy Institute for Biomedical Sciences, Zewail City of Science, Technology and Innovation, October Gardens, Giza, 12578 Egypt; 3 Institute of Global Health and Human Ecology, American University in Cairo, New Cairo, 11835 Egypt; 4 Department of Biology, School of Sciences and Engineering, American University in Cairo, AUC Ave, P.O. Box 74, 11835 New Cairo, Egypt

**Keywords:** Spinal cord injury, SCI, Differentially expressed genes, DEGs, Regeneration, Gene expression, Microarray, RNAseq, Meta-analysis

## Abstract

The limited regenerative capacity of human nervous tissue compared to other species has long fascinated researchers. Remarkably, non-mammalian vertebrates such as teleost fish and axolotls possess robust regenerative abilities, capable of regenerating entire limbs and even fully transected spinal cords. Understanding the cellular and molecular mechanisms governing spinal cord regeneration across species may offer insights to accelerate repair processes and improve the quality of life for spinal cord injury (SCI) patients. This systematic review and meta-analysis compiled genome-wide spinal cord gene expression datasets from published SCI studies spanning multiple species. A targeted search was conducted using Gene Expression Omnibus, PubMed, and Science Direct, yielding 167 studies employing microarray and RNA sequencing (RNA-seq) technologies. After screening, 42 studies were selected: 20 used microarray, 17 used bulk RNA-seq, 2 employed single-cell RNA-seq (scRNA-seq), 2 combined bulk and scRNA-seq, and 1 incorporated both single-nucleus RNA-seq (snRNA-seq) and scRNA-seq. Approximately 43% of the selected studies were performed on *Rattus norvegicus*. Nine studies met the criteria for meta-analysis, allowing for cross-species comparison. Differentially expressed genes (DEGs) varied across models, reflecting species-specific responses. At day 7 post-injury, 214 DEGs were shared between regenerative (REG) and non-regenerative (non-REG) species. Across acute and subacute phases (multiple time points within the first week), 694 shared DEGs were identified. A month post-injury, 51 genes overlapped. Overall, 824 DEGs were common to the REG and non-REG groups at all time points. To explore functional relationships, protein–protein interaction (PPI) analysis was conducted on the oppositely regulated DEGs shared between REG and non-REG groups. Using Cytoscape and the CytoHubba plugin, key hub genes were identified, including CCNA2, CCNB1, CCNB2, CDC20, and FBXO5, all of which are associated with cell cycle processes. These hub genes were upregulated in non-REG (poorly regenerating) mouse models and may influence regenerative divergence across species by modulating glial proliferation or scar formation. Enrichment analysis revealed significant activation of cell cycle and mitotic processes in the non-REG group, which suggests cell cycle progression in response to injury.

**Supplementary Information:**

The online version contains supplementary material available at 10.1007/s12035-025-05496-y.

## Introduction

Human spinal cord injury (SCI) is a severe condition with potentially devastating outcomes that range from loss of sensation and paralysis to even death [1]. The main cause of SCI can be attributed to accidents in the youth or young working-age individuals. This represents a remarkable economic burden on societies due to expenses being spent on rehabilitation and overall therapy [2]. It was reported that SCI affects almost 54 cases per million individuals, or nearly 17,000 new cases each year in the United States [3]. Globally, according to the World Health Organization, it was estimated that between 250,000 and 500,000 individuals suffer from SCI every year [4]. In spite of having a substantial impact on patients’ lives and preclinical achievements in regenerative medicine, till now, regeneration of the spinal cord is unachievable in humans, and to date, none of these preclinical advances has led to an effective treatment for SCI on the clinical level. Only methylprednisolone is approved for SCI therapy (within 8 h of trauma). The aim of therapies is to reduce the inflammation as much as possible in order to prevent the spread and progression of damage from reaching more areas of the spinal cord tissue and to enhance regeneration [5].

Notably, mammals, especially humans, have limited tissue regeneration capabilities in comparison to other species [6, 7]. After SCI, axolotls achieve complete nervous tissue restoration, motor, and sensory function recovery, together with restoration of dorsal root ganglia and the ependymoglial lining [8]. Within the 48 h after SCI, zebrafish larvae show rapid axonal reconnection across the site of the spinal cord lesion, together with full restoration of motor function as confirmed by swimming behavior assessments [9]. 

*Xenopus tropicalis* tadpoles exhibit spinal cord regeneration during early stages of development [10]. In contrast, in rodents, SCI results in permanent loss of function and formation of glial scars (F. [11]).

SCI can be defined as the damage that occurs in the spinal cord that leads to disruption of motor function of body parts and as well as sensation below the site of injury. SCI starts with a primary injury, and then, it proceeds and progresses to what is called secondary injury. Primary injury results in direct damage to the spinal cord tissue, leading to cut axons and broken blood vessels. Secondary injury starts within minutes of the occurrence of the primary injury. Secondary injury later on results in the spread of the injury and limits restorative processes. Resulting damage includes reduced blood flow to the spinal cord, hemorrhage, apoptosis, and production of inflammatory mediators, as well as free radicals. This cascade leads to complications that continue to expand in both directions of the injury along the spinal cord for up to 72 h, increasing the damage even more. That is why initial management of SCI is most concerned with the prevention of progression to secondary injury [12, 13].

To understand the mechanisms underlying spinal cord regeneration, many studies analyzed differentially expressed genes (DEG) following SCI. Some previous reviews and comparative studies examined gene expression across two or more species following SCI. Among these studies was one that conducted a comparison between only two species (rat and axolotl), which relied exclusively on RNA-seq data. Authors focused on inflammatory and extracellular matrix remodeling gene expression between rat and axolotl [14]. Another study conducted cross cross-species comparison after SCI across three organisms (rats, mice, and salamanders) and was restricted to microarray datasets [15]. The study suggested the MAPK signaling pathway as a potential contributor to poor regeneration in rodent models tested. In contrast, our study integrates both microarray and RNA-seq studies and focuses on shared oppositely regulated DEGs after SCI and emphasizes comparative hub gene analysis. This sheds light on gene expression patterns potentially associated with regenerative ability, providing a wider comparative perspective that is currently lacking.

## Methods

### Search Strategy

**Fig. 1:**
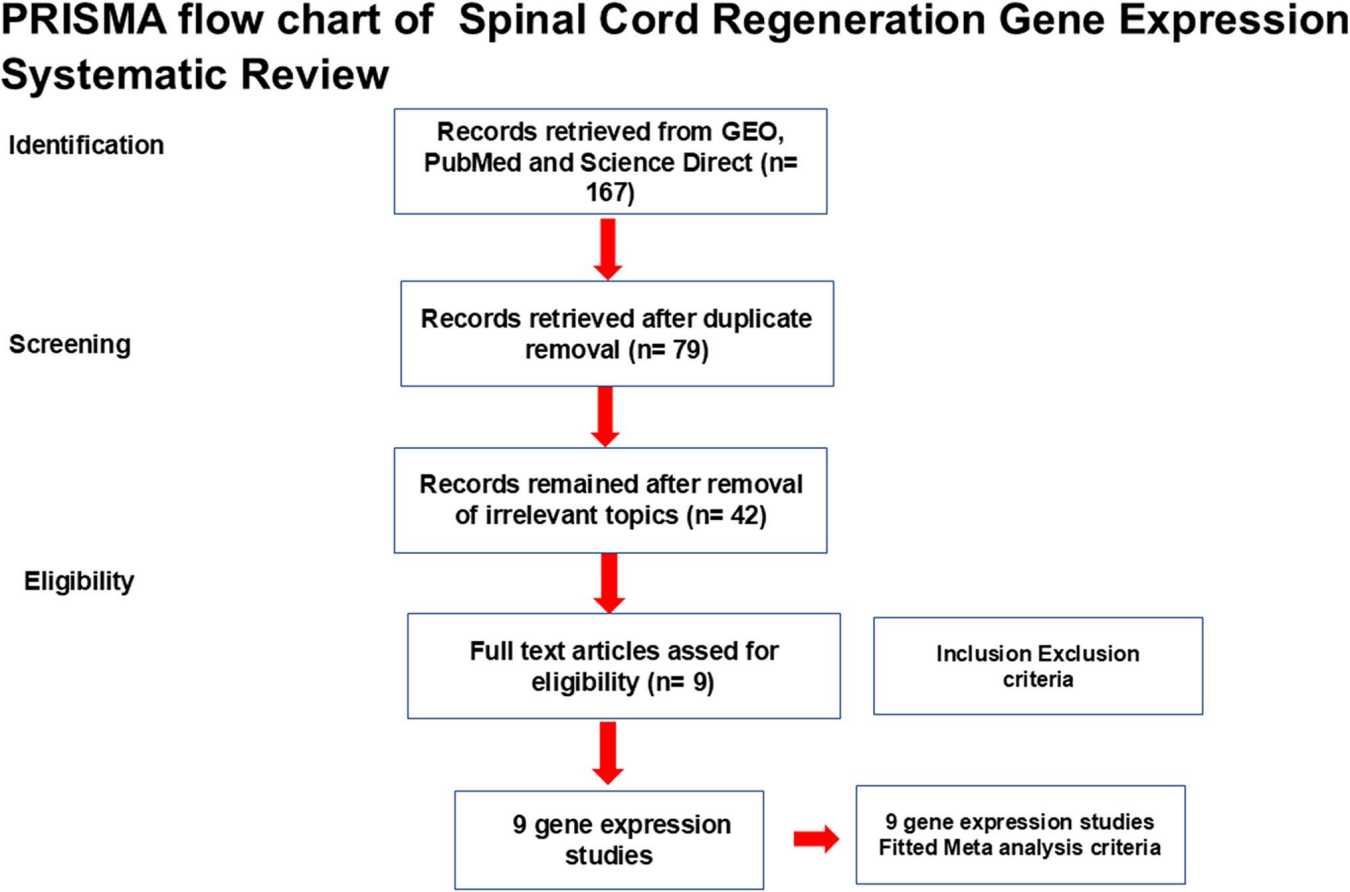
PRISMA flowchart of RNA Seq Spinal Cord Regeneration Gene Expression Systematic Review

The systematic review and meta-analysis were performed according to the guidelines of the Preferred Reporting Items for Systematic Reviews and Meta-Analyses (PRISMA) [16]. The electronic search was performed on studies (published between January 2000 and December 2023) in PubMed based on related Medical Subject Headings (MeSH) terms: “Gene Expression” AND “Spinal cord injury” AND “Regeneration.” In Gene Expression Omnibus (GEO), the search was performed using terms like “Transcriptome,” “Spinal cord injury”, AND “Regeneration”, “differentially expressed genes.” On Science Direct, the search was performed using the terms: “Gene Expression,” “Spinal Cord regeneration,” “microarray,” and “RNA seq.” A PRISMA flow chart for our search is shown in Fig. 1.

### Systematic Review

One hundred eight studies were collected and identified from this search and screened for eligibility based on 4 inclusion criteria and 2 exclusion criteria. Inclusion criteria were (1) published in English, (2) genome-wide analysis, (3) Differential Gene Expression Studies, and (4) studies of spinal cord tissue samples collected from SCI models. Exclusion criteria are (1) non-gene expression studies and (2) studies of tissues other than the spinal cord.

Furthermore, studies were excluded when the data were not reported, the data were unclear, the study was not genome-wide, the study was not relevant, or the study’s supplementary material was not provided.

Studies that met all 6 criteria were 42 and were therefore included in the systematic review.

### Meta-analysis

Studies were included in the meta-analysis if they met 3 additional criteria: (1) non-intervention studies (no treatment), (2) original dataset studies, (3) DEG list is provided. Intervention studies were excluded from our study to ensure that comparisons of DEG reflect the natural biological responses to SCI, rather than any effects influenced by genetic or pharmacological manipulations.

Of the 42 studies included in our systematic literature review, 9 gene expression studies met our meta-analysis criteria and were further analyzed. All reported significant DEGs based on each study’s significance threshold for differential expression were selected.

DEGs were classified as upregulated or downregulated based on their reported log₂ fold change (log₂FC) values. Upregulated and downregulated genes were sorted for each dataset from the provided log2-fold change. A log₂FC cutoff was applied. Genes with log₂FC ≥ 1 or ≤ –1 were considered differentially expressed. Up to the top 500 upregulated together with the top 500 downregulated DEGs were selected for 9 studies to make a total of 1000 DEGs for each study. This step combines both fold change filtering and rank-based selection, ensuring that selected DEGs met the significance thresholds as reported in their respective studies (typically *p* < 0.05). Two studies out of the nine studies [17] and [18] provided fold change. Log2 fold change values were calculated manually using Microsoft Excel, and the top 1000 DEGs were extracted. Significant *p*-values provided were sorted. Overlap was determined by the presence of the same gene in the gene lists from different studies.

For pathway enrichment, GeneCodis4 and g:Profiler were used, applying the Benjamini–Hochberg false discovery rate (FDR) correction method, with an adjusted *p*-value (FDR) < 0.05 as the significance threshold. The two tools were used complementarily to ensure the reproducibility of functional annotation results. Enrichment was performed on each study’s DEG list separately using standard organism-specific background according to the Kyoto Encyclopedia of Genes and (KEGG, www.kegg.jp/kegg/kegg1.html) database. In GeneCodis4, the statistical significance of each annotation or set of concurrent annotations was calculated based on its frequency in the input and reference sets. Due to the absence of annotated reference data for *A. mexicanum*, this species was excluded from the enrichment analysis.

Bar charts were generated using Microsoft Excel. Venn diagrams were generated using Good Calculators (goodcalculators.com/venn-diagram-maker/).

### Protein–Protein Interaction (PPI) Network Construction and Hub Gene Identification

To explore functional interaction, PPI interaction networks were conducted using StringDB-v10.5 [19]. Shared oppositely regulated DEGs between REG and non-REG groups during the acute/subacute phase post-injury (1, 2, 3, and 7 days post-injury) were used as input representing the initial response phase after SCI (DEG list is provided in supplementary data 4). This early phase is suggested to be critical for determining the trajectory of repair, as it includes inflammation and signaling cascades that may either promote regeneration or lead to scar formation. Shared oppositely regulated DEGs have been matched to human orthologs. *Homo sapiens* was chosen as the reference species in STRING due to the comprehensive functional interaction data available across species, ensuring consistency. CytoScape-v3.6 was used for visualization [20] using a medium confidence score cutoff of 0.4. Disconnected nodes were excluded. Hub genes were identified by the CytoHubba plugin based on Maximal Clique Centrality (MCC). Functional enrichment analysis was conducted in Cytoscape to explore associated Gene Ontology Biological Processes (GO: BP) and KEGG pathways using the ClueGO plugin.

## Results

### Systematic Review

Out of the 167 studies identified (108 in GEO, 9 in PubMed, and 50 in Science Direct), 42 gene expression studies of spinal cord tissue samples from 8 species (*A. mexicanum*, *D. rerio*, *M. musculus*, *R. norvegicus*, *P. marinus*, *M. domestica*, *M. mulatta*, *X. tropicalis*) were eligible for the systematic review (Table 1). All studies were published between April 2007 and August 2023. Of these 167 studies, 20 studies used microarray, 17 studies used bulk RNA seq, 2 studies used both bulk RNA seq and SC RNA seq, 2 studies used SC RNA seq, and 1 study used SN RNA seq and SC RNA seq (supplementary data 1a).

**Table 1:** List of studies used in the systematic review

Species	Intervention	Approach
Axolotl, *Ambystoma mexicanum*	None	Microarray [6]
Axolotl, *Ambystoma mexicanum*	None	Microarray [21]
Frog, *Xenopus tropicalis*	None	RNA-seq [22]
Lamprey, *Petromyzon marinus*	None	RNA-Seq [23]
Monkey, *Macaca mulatta*	None	SN RNA seq and SC RNA-Seq [24]
Mouse, *Mus musculus*	EphA4 knockout	Microarray [25]
Mouse, *Mus musculus*	None	RNA-Seq (K. [17])
Mouse, *Mus musculus*	Transgenesis (GFAP-IκBα-dn mice)	Microarray [26]
Mouse, *Mus musculus*	None	RNA-Seq [27]
Mouse, *Mus musculus*	Il1a-knockout and Il1b-knockout	Microarray [28]
Mouse, *Mus musculus*	Knock out (Arntl/Bmal1)	RNA-Seq [29]
Mouse, *Mus musculus*	Gsx1 gene therapy	RNA-Seq [30]
Mouse, *Mus musculus*	None	RNA-seq (Y. [31])
Mouse, *Mus musculus*	Interleukin-4 and interleukin-13 injections	RNA-Seq [32]
Mouse, *Mus musculus*	Downregulating circular RNA Prkcsh	RNA-Seq (J.-N. [33])
Mouse, *Mus musculus*	None	SC RNA-Seq (J. [34])
Mouse, *Mus musculus*	Genetically reducing expression levels of synj1	RNA-Seq [35]
Mouse, *Mus musculus*	None	RNA-Seq [36]
Mouse, *Mus musculus*	Caloric restriction mimetics (3,4-dimethoxychalcone)	RNA-Seq [37]
Opossum, *Monodelphis domestica*	None	RNA-Seq [38]
Rat, *Rattus norvigecus*	None	Microarray [39]
Rat, *Rattus norvegicus*	Mesenchymal stem cells or olfactory ensheathing cells transplantation	Microarray [40]
Rat, *Rattus norvegicus*	Electrical stimulation	Microarray (Y. [31])
Rat, *Rattus norvegicus*	None	RNA-Seq [18]
Rat, *Rattus norvegicus*	High-fat diet-induced obesity	Microarray [41]
Rat, *Rattus norvegicus*	Albumin-hydroxy oleic acid complex administration	Microarray [42]
Rat, *Rattus norvegicus*	None	Microarray [43]
Rat, *Rattus norvegicus*	Treadmill training	RNA-Seq [44]
Rat, *Rattus norvegicus*	Ketogenic diet	Microarray [45]
Rat, *Rattus norvegicus*	Intrathecal transplantation of human umbilical cord-derived mesenchymal stem cells	RNA-Seq (T. [46])
Rat, *Rattus norvegicus*	Erxian decoction [47]	RNA-Seq [47]
Rat, *Rattus norvegicus*	Neural progenitor cell transplantation	Microarray [48]
Rat, *Rattus norvegicus*	Hyperbaric oxygen therapy	Microarray [49]
Rat,* Rattus norvegicus*	Hepatocyte growth factor-releasing scaffold and human iPS cell-derived neural stem/progenitor cells	RNA-Seq [50]
*Rattus norvegicus*	None	Microarray (J. [51, 52])
*Rattus norvegicus*	None	Microarray (J. [51, 52])
*Rattus norvegicus*	Methylprednisolone	Microarray [53]
*Rattus norvegicus*	Treadmill training	Microarray [54]
Zebra fish, *Danio rerio*	None (bulk RNA seq), Knock down (single cell) None (methylation)	RNA-Seq and SC RNA-Seq [55]
Zebra fish, *Danio rerio*	None	Microarray [56]
Zebra fish, *Danio rerio*	None	RNA-Seq (C.-X. [57])
Zebra fish, *Danio rerio*	None	Microarray [58]

**Fig. 2:**
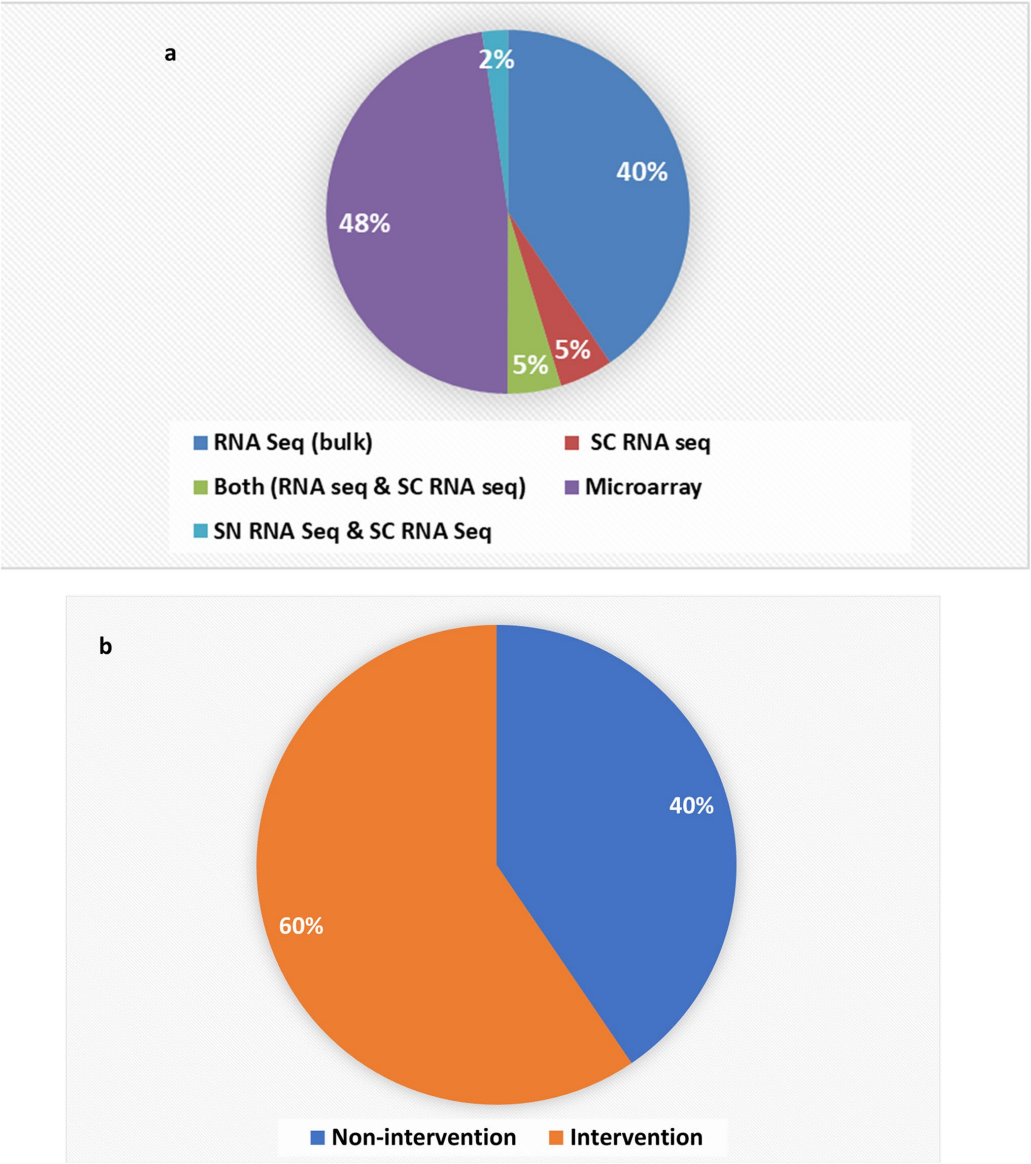
**Studies included in the analysis.** **a** Pie chart showing the percentage of studies vs. the gene expression approach used. **b** Pie chart showing intervention studies vs. non intervention studies. Most of the studies included were intervention studies (~ 60%)

Microarray studies (47.6%) were followed by RNA seq studies (40.4%), forming the majority of the studies (Fig. 2a and supplementary data 1b). Studies with interventions (59.5%) were higher than non-intervention studies (40.47%) (Fig. 2b and supplementary data 1c).

**Fig. 3:**
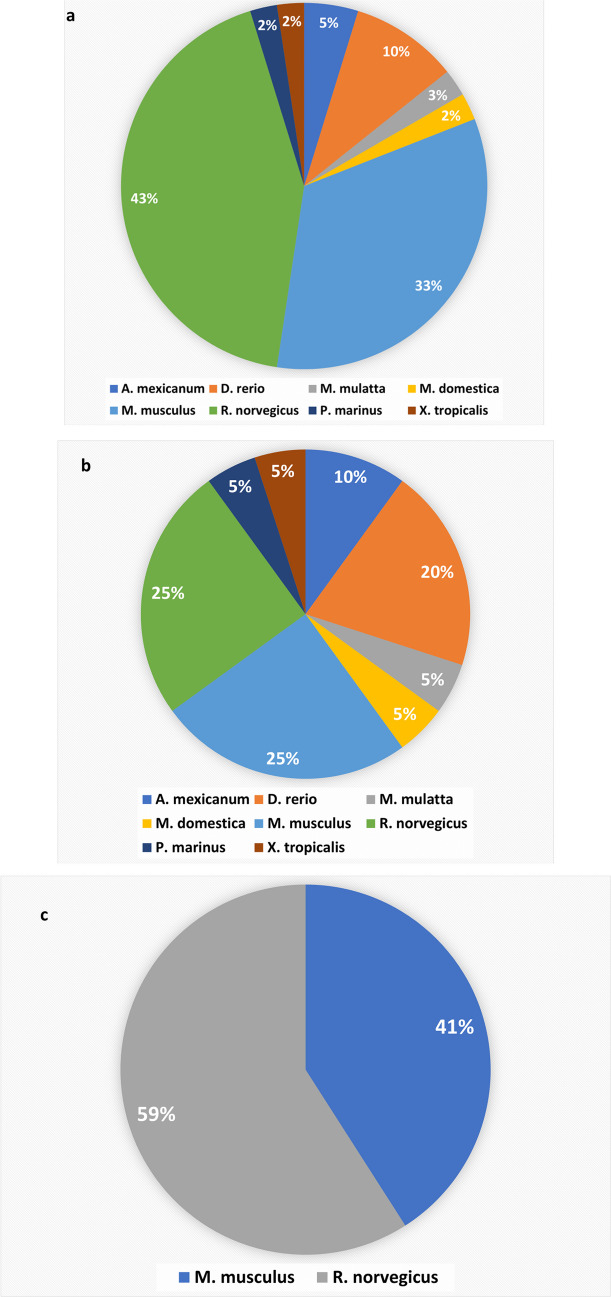
**Studies included in the analysis vs. species**. **a** Pie chart showing the percentage of all studies (intervention and non-intervention) vs. species. *Rattus norvegicus* (43%) was the species with the highest percentage in studies used in the systematic review. **b** *Rattus norvegicus* and *M. musculus* studies represented the highest percentage in non-intervention studies. **c** Intervention studies included *R. norvegicus* and *M. musculus* studies

The current systematic review identified 42 studies on 8 species. Nearly 43% (18 studies) of all studies were performed on rats (*R. norvegicus*) (Fig. 3a and supplementary data 1 d). Moreover, *R. norvegicus* (25%) and *M. musculus* (25%) studies represented the highest percentage in non-intervention studies (Fig. 3b and supplementary data 1e). Intervention studies included *R. norvegicus* (59%) and *M. musculus* (40.9%) (Fig. 3c and supplementary data 1f).

### Meta-analysis

The meta-analysis of 9 gene expression studies was performed across 6 species (*A. mexicanum*, *D. rerio*, *M. musculus*, *P. marinus*, *R. norvegicus*, and *X. tropicalis*)*.* Non-REG species included *M. musculus* and *R. norvegicus*, while REG species included *A. mexicanum*, *D. rerio*, *P. marinus*, and *X. tropicalis*. Studies selected for meta-analysis are listed in Table 2.

**Table 2:** List of 9 studies included in the meta-analysis

Species	Organism	Time points after SCI	Approach
*A. mexicanum*	Axolotl	Days 1, 3, 5, and 7	Microarray [6]
*M. musculus*	Mouse	Days 2 and 7	RNA-Seq (K. [17])
*P. marinus*	Lamprey	Days 1,3, 7, and 28	RNA-Seq [23]
*D. rerio*	Zebra fish	Days 1, 3, and 7	Microarray [58]
*M. musculus*	Mouse	Day 3 & Day 7	RNA-Seq [27]
*R. norvegicus*	Rat	Day 30	RNA-Seq [18]
*M. musculus*	Mouse	Days 2, 7, and 28	RNA-seq [59]
*X. tropicalis*	Frog tadpole	Days 1 and 3	RNA-seq [22]
*D. rerio*	Zebra fish	Day 7	RNA-Seq and SC RNA-Seq [55]

#### Differentially Expressed Genes (DEG) at Day 7 Post-injury

The comparison of DEG at day 7 post-injury (pi) included 4 species (*A. mexicanum*, *D. rerio*, *M. musculus*, and *P. marinus*).

**Fig. 4:**
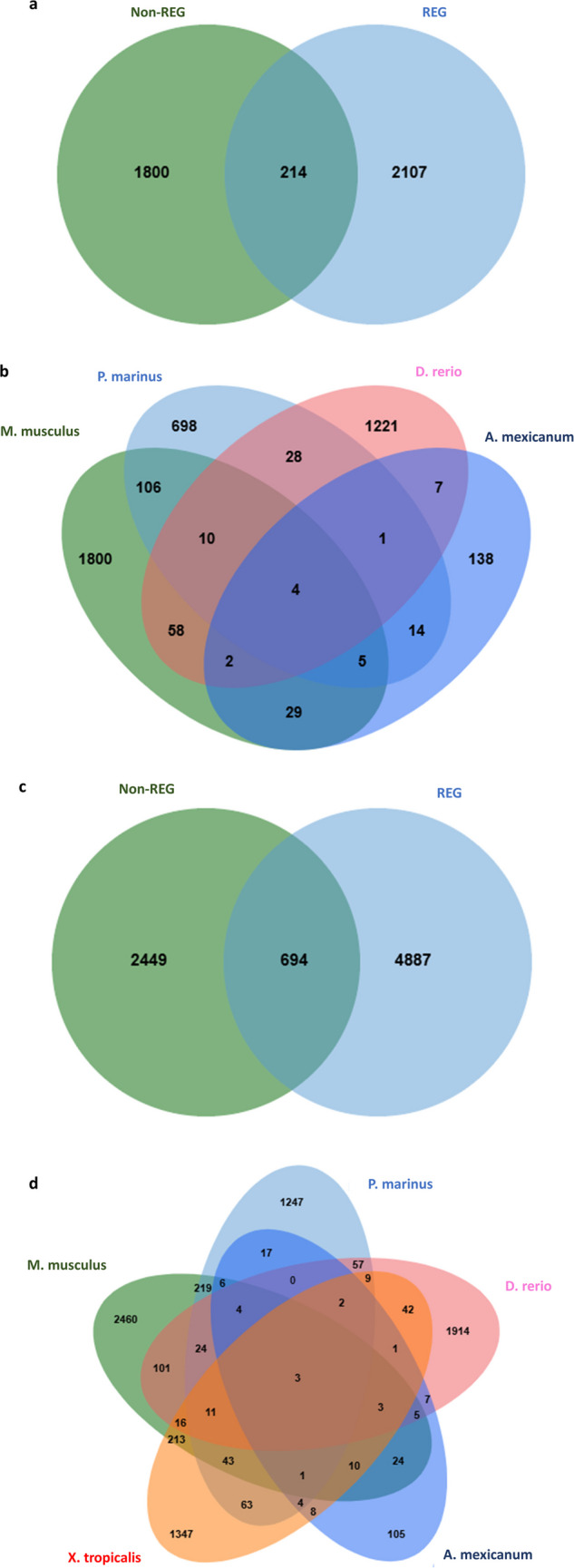
**Overlapping DEG at day 7 and during week 1 after SCI injury.** **a** Venn diagram illustrating shared differentially expressed genes (DEGs) between non-regenerating (non-REG; *Mus musculus*) and regenerating (REG; *Ambystoma mexicanum*, *Danio rerio*, and *Petromyzon marinus*) species at day 7 post-spinal cord injury (SCI). **b** Venn diagram showing overlapping DEGs among the four species (*M. musculus*, *A. mexicanum*, *D. rerio*, *P. marinus*) on day 7 post-SCI. **c** Venn diagram depicting shared DEGs between non-REG (*M. musculus*) and REG species (*A. mexicanum*, *D. rerio*, *P. marinus*, and *Xenopus tropicalis*) during the first week after SCI (days 1, 2, 3, and 7). **d** Venn diagram showing common DEGs across all five species on days 1, 2, 3, and 7 following SCI

A total of 214 DEG overlapped between the REG group (*A. mexicanum*, *D. rerio*, and *P. marinus*) and the non-REG group (*M. musculus*) (Fig. 4a). Shared DEG between each species is shown in Fig. 4b. Lists of overlapped gene lists are provided in the supplementary data 2a.

#### Differentially Expressed Genes (DEG) After Week 1 Post-Injury (All Week 1 Time Points)

The comparison of DEG at all time points during the first week after injury (days 1, 2, 3, 5, and 7) included 5 species (*A. mexicanum*, *D. rerio*, *M. musculus*, *P. marinus*, and *X. tropicalis*).

**Fig. 5:**
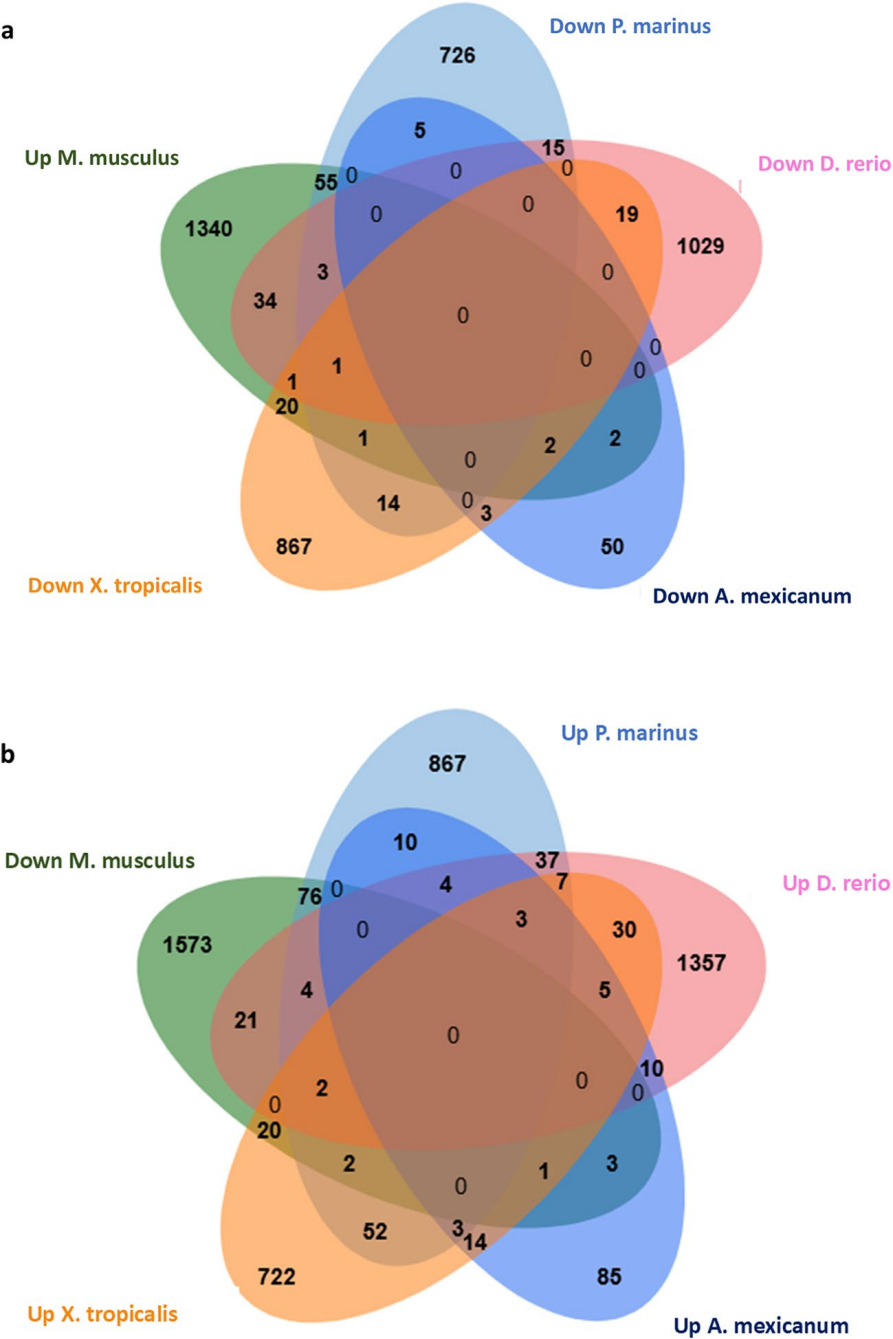
**Shared oppositely regulated DEGs between** **non-REG and REG species during the first week after SCI injury**. **a** Venn diagram displaying overlapping genes that are upregulated in the non-regenerating species (*Mus musculus*) and downregulated in regenerating species (*Ambystoma mexicanum*, *Danio rerio*, *Petromyzon marinus*, and *Xenopus tropicalis*) during the first week following spinal cord injury (SCI). **b** Venn diagram showing genes that are downregulated in *M. musculus* and upregulated in the regenerating species listed above during the same post-injury timeframe

When all week 1 time points were pooled, a total of 694 genes overlapped between the non-REG group (*M. musculus*) and the REG group (*A. mexicanum*, *P. marinus*, *D. rerio*, and *X. tropicalis*) (Fig. 4c). Shared DEG between each species is shown in Fig. 4d. Oppositely regulated DEGs during the first week after injury between species are shown in Fig. 5a and b. Lists of all shared and oppositely regulated DEGs are provided in the supplementary data 2b and 2c, respectively.

#### Differentially Expressed Genes (DEGs) at Day 28/30 Post-Injury

**Fig. 6:**
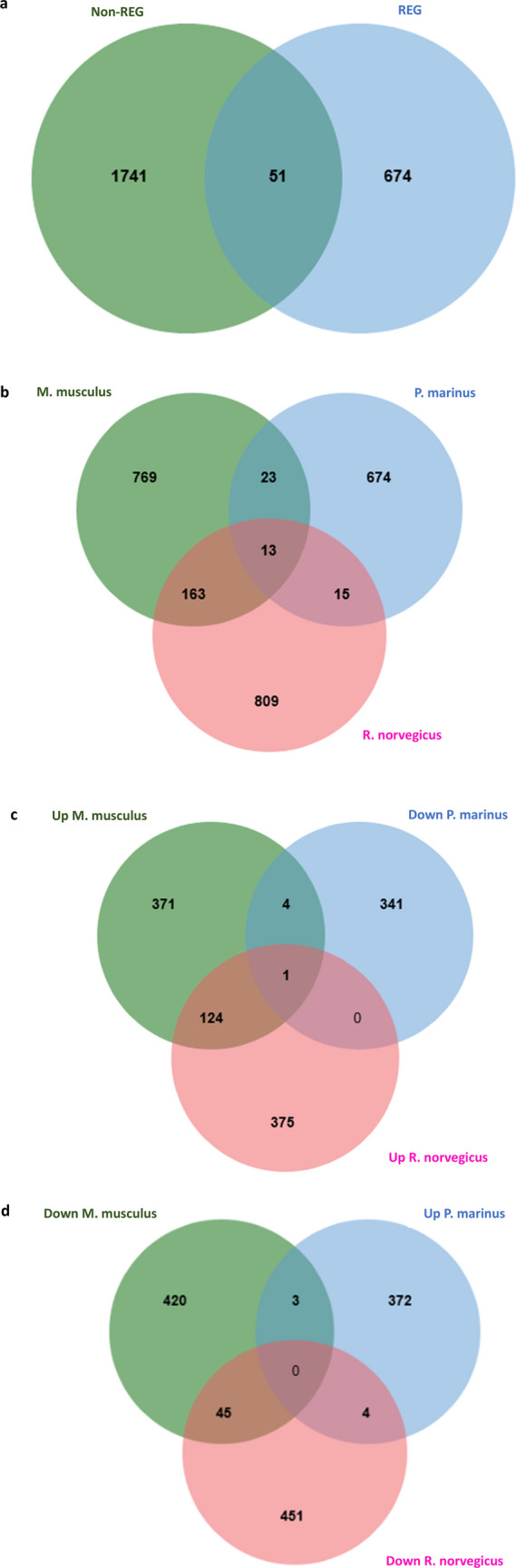
**Overlapping and oppositely regulated DEGs in different species at one month after SCI**. **a** Venn diagram showing shared differentially expressed genes (DEGs) between non-regenerating species (*Mus musculus* and *Rattus norvegicus*) and the regenerating species (*Petromyzon marinus*) at four weeks post–spinal cord injury (SCI). **b** Venn diagram depicting DEGs commonly expressed across all species tested 1 month after SCI. **c** Venn diagram highlighting genes that are upregulated in non-regenerating species (*M. musculus* and *R. norvegicus*) and downregulated in the regenerating species (*P. marinus*) within the same time frame. **d** Venn diagram illustrating genes that are downregulated in *M. musculus* and *R. norvegicus*, and upregulated in *P. marinus* at 1 month post-injury

The comparison of DEG at 4 weeks/28 days/1 month pi included 3 species (*M. musculus*, *P. marinus*, and *R. norvegicus*). Fifty-one shared DEG overlapped between the non-REG group (*M. musculus* and *R. norvegicus*) and the REG group (*P. marinus*) (Fig. 6a). Shared DEG between each species is shown in Fig. 6b. Shared oppositely regulated DEG between each species are shown in Fig. 6c and d. List of shared and oppositely regulated DEGs is provided in the supplementary data 2d and 2e, respectively.

#### Differentially Expressed Genes (DEGs) from All Time Points Combined

**Fig. 7:**
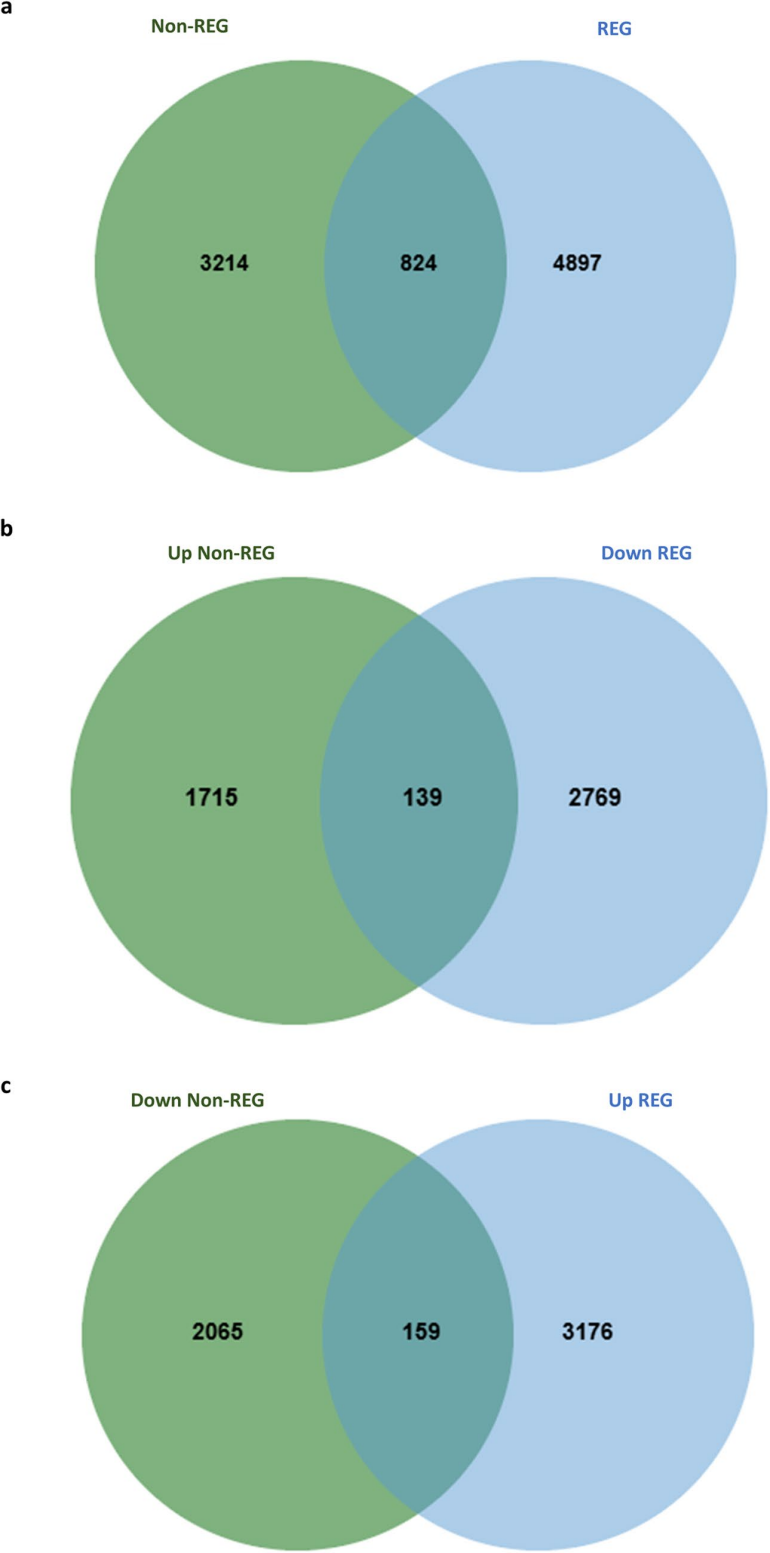
**Overlapping DEG and oppositely regulated at all time points**. **a** Venn diagram showing overlapping differentially expressed genes (DEGs) between non-regenerating species (*Mus musculus* and *Rattus norvegicus*) and regenerating species (*Ambystoma mexicanum*, *Danio rerio*, *Petromyzon marinus*, and *Xenopus tropicalis*) across all post-injury time points. A total of 824 genes were commonly expressed between the two groups. **b** Venn diagram illustrating 139 DEGs that were upregulated in the non-regenerating species (*M. musculus* and *R. norvegicus*) and downregulated in the regenerating species. c) Venn diagram highlighting 159 genes that were downregulated in the non-regenerating species and upregulated in the regenerating species during the same injury period

When DEGs from all time points (day 1, 2, 3, 7, and 28/30) pi were merged, 824 genes were shared between non-REG group (*M. musculus* and *R. norvegicus*) and REG group (*A. mexicanum*, *P. marinus*, *D. rerio*, and *X. tropicalis*) (Fig. 7a and supplementary data 2f). 139 DEGs were upregulated in the non-REG group while downregulated in the REG group (Fig. 7b). 159 DEG were downregulated in the non-REG group and upregulated in the REG group (Fig. 7c). Lists of oppositely regulated DEGs are provided in the supplementary data 2 g and 2 h, respectively.

Kyoto Encyclopedia of Genes and Genomes (KEGG) database search indicated that in all species DEG included in our study (except for *A. mexicanum*, which does not have annotation data), there is a large enrichment in genes associated with cell cycle, DNA replication, neuroactive ligand-receptor interaction, metabolic pathways, apoptosis, purine metabolism, and pyrimidine metabolism. Non-REG species, *M. musculus*, and *R. norvegicus* DEG were enriched for pathways of complement and coagulation cascades, neurodegeneration, inflammatory mediator regulation of TRP channels, IL-17 signaling pathway, tumor necrosis factor signaling pathway, cAMP signaling pathway, and PI3K-Akt signaling pathway (supplementary data 3).

### Protein–Protein Interaction (PPI) Network and Hub Gene Identification

**Fig. 8:**
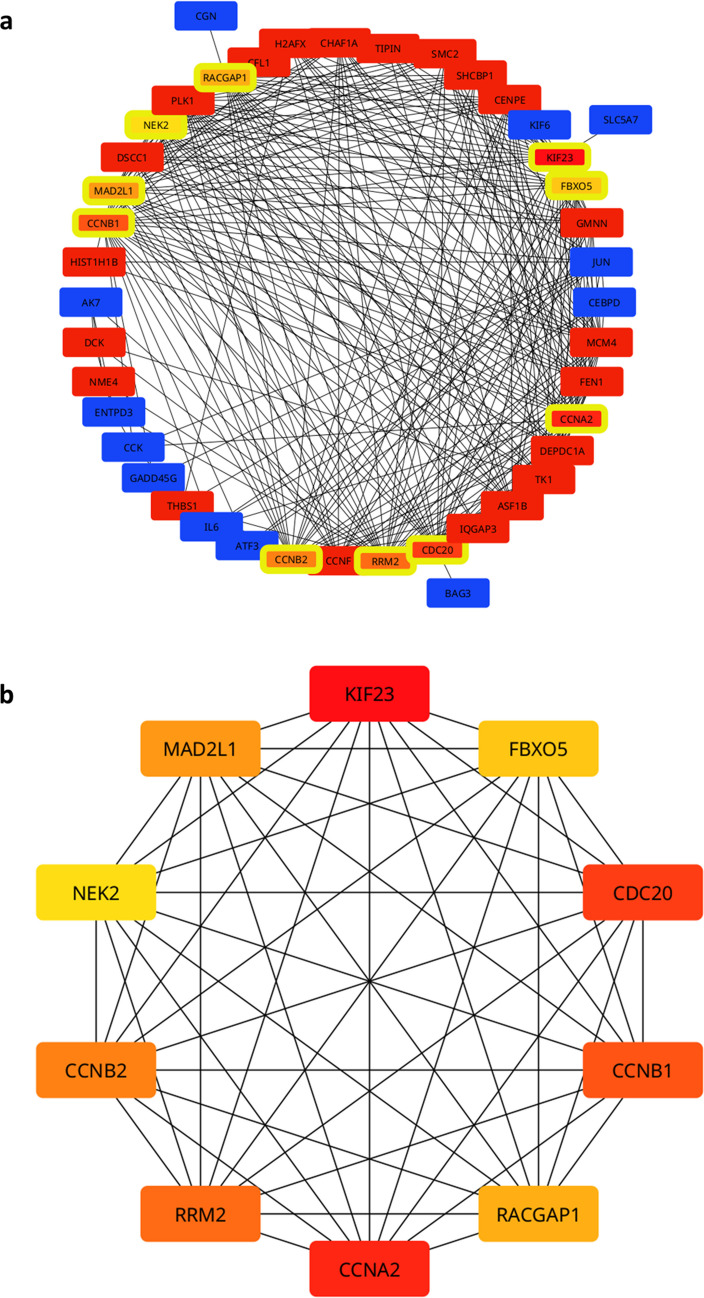
**Protein–protein interaction (PPI) network**. **a** Protein–protein interaction (PPI) network illustrating shared differentially expressed genes (DEGs) in the non-regenerating species (*Mus musculus*). Red nodes represent upregulated shared DEGs, blue nodes indicate downregulated shared DEGs, and nodes with yellow borders highlight screened hub genes. **b** Hub genes identified using maximal clique centrality (MCC) scoring. All displayed hub genes were upregulated in the non-regenerating group and downregulated in the regenerating species

The constructed PPI network identified upregulated and downregulated DEGs in mice, together with central hub genes (Fig. 8a). The top 10 hub genes identified were CCNA2 CCNB1, CCNB2, CDC20, FBXO5, KIF23, MAD2L1, NEK2, RACGAP1, and RRM2 (Fig. 8b). KEGG pathway enrichment analysis of upregulated genes in the non-REG group (*M. musculus*) revealed significant activation of the cell cycle pathway (Table 3). Gene Ontology Biological Process (GO:BP) terms enriched among the hub genes included mitotic cell cycle process, cell cycle process, mitotic sister chromatid segregation, positive regulation of fibroblast proliferation, microtubule cytoskeleton organization involved in mitosis, and positive regulation of cell population proliferation (Table 4). These processes are associated with cell cycle progression and proliferative responses. Several hub genes driving these enrichments included CCNA2, CCNB1, CCNB2, CDC20, and MAD2L1 (for cell cycle), and CCNA2, CCNB1, CCNB2, KIF23, CDC20, NEK2, MAD2L1, and RACGAP1 (mitotic cell cycle process). Further enrichment details are provided in the supplementary data 5.

**Table 3:** Enriched KEGG pathways in the non-REG group compared to the REG group during week one after SCI

KEGG pathway	FDR value
Neuroactive ligand-receptor interaction	3.50E-04
Protein digestion and absorption	3.50E-04
Cell cycle	0.0209
p53 signaling pathway	0.0333
Pertussis	0.0333
Pyrimidine metabolism	0.0474

**Table 4:** Enriched GO: BP in the non-REG group compared to the REG group during week one after SCI

Go: BP	FDR value
Mitotic sister chromatid segregation	0.0021
Positive regulation of fibroblast proliferation	0.003
Positive regulation of cell population proliferation	0.0248
Mitotic cell cycle process	0.0267
Nuclear division	0.0374
Mitotic spindle organization	0.0387
Cell cycle process	0.0475

## Discussion

Despite tens of millions of years of evolutionary divergence, it was suggested that gene expression was more similar between homologous tissues of different species than between different tissues of the same species [60]. Therefore, we conducted a comparison of the gene expression differences of spinal cord tissue after SCI among different species to explore conserved genes that are associated with enhanced neuronal regeneration.

We conducted a systematic review of the literature that included 8 species (*A. mexicanum*, *D. rerio*, *M. domestica*, *M. mulatta*, *M. musculus*, *P. marinus*, *R. norvegicus*, and* Xenopus tropicalis*) as well as a meta-analysis for 6 species (*A. mexicanum*, *D. rerio*, *M. musculus, P. marinus*, *R. norvegicus*, and *X. tropicalis*).

First, based on our systematic review eligibility criteria of the research papers, 42 out of 167 research papers were selected for further analysis. This is because most of the 167 research papers included intervention (treatment, transgenesis, gene knock out, gene silencing, etc.), which did not fit our inclusion/exclusion criteria. In addition, studies were found to adopt variant guidelines regarding data availability. For example, some studies provided a list of the involved pathways, while others did not provide these lists. The unavailability of a standard way of data reporting made it challenging to use some data in our review. We also consider that the starting number of papers (167) is relatively small. This highlights the need for more gene expression studies on spinal cord regeneration in the near future.

Only 9 studies were found eligible for our meta-analysis inclusion and exclusion criteria. One possible explanation may be the difficulty in collecting data having the same time points and the same variables. Another reason is that these 9 papers are the only ones that provide DEG lists. Accordingly, taking into consideration all these factors, matching data from different samples was challenging.

The number of overlapped DEG from week 1 cross-species comparison was much higher than that of week 4 comparison (694 vs. 51). Among the reasons for this is that most of the DEG lists included in the week 1 comparison provided multiple acute or subacute phase time points (from day 1 to day 7 post-injury). On the other side, week 4 comparison included one time point (day 30). *Rattus norvegicus* day 30 pi list was the only DEG list recruited, as earlier time points were not provided by the original study [18]. Finally, the day 30/week 4 comparison included only 3 species (*M. musculus*, *P. marinus*, and *R. norvegicus*) vs. 5 species that were included in week 1 comparison (*A. mexicanum*, *D. rerio*, *M. musculus*, *P. marinus*, and* X. tropicalis*).

In pursuit of identifying conserved yet divergent molecular responses across multiple species with different regenerative abilities, the study focused on shared DEGs that were oppositely regulated between non-REG and REG groups during the first week after SCI. This spans the acute/subacute phase, which is critical in determining either regenerative or fibrotic outcomes. By using these DEGs as input in the STRING database for PPI analysis, our aim was to identify key hub genes that were consistently shared, yet differently regulated, in the regenerating versus non-regenerating context. This allows us to unravel significant interaction networks that might be lost when focusing solely on similarly regulated DEGs. Moreover, STRING incorporates both known and predicted interactions based on orthology, allowing it to be highly suitable to uncover conserved functional interactions across different species.

Notably, all identified hub genes were upregulated in the non-REG group (*M. musculus*) while downregulated in at least one species in the REG group (*A. mexicanum*, *D. rerio*, *P. marinus*, and *X. tropicalis*) during week one after SCI. Several of the identified hub genes, including CCNA2, CCNB1, CCNB2, CDC20, and FBXO5, were suggested among regulators of the cell cycle and were shown to contribute to glial proliferation and scar formation following injury of the CNS. Persistent cell cycle activation in post-mitotic neurons or reactive astrocytes was also suggested to be a core attribute of the failed regenerative response observed in adult mammals. Particularly, cyclins (CCNA2 and CCNB1) were upregulated in astrocytes and were associated with glial scar formation, which poses a major barrier to axonal regrowth [61]. Similarly, CDC20 and FBXO5 contribute to mitotic progression [62, 63]. Cell cycle genes’ persistent expression might sustain proliferative glial states, worsening tissue remodeling and functional repair [64]. Together, our results support the hypothesis that aberrant or prolonged cell cycle activation is a key feature of the non-permissive environment post-SCI and emphasize the potential of targeting these genes or their pathways in order to enhance repair.

Persistent cell cycle activation in mice observed in enriched KEGG and GO terms is indicative of aberrant re-entry into mitosis by post‑mitotic cells, including neurons and glia. This maladaptive response was linked to apoptosis, glial proliferation, and poor regeneration [61, 65]. In parallel to cell cycle activation, upregulation of fibroblast-related GO terms (e.g., positive regulation of proliferation) likely indicates fibrotic scar formation, which is mediated by activated fibroblasts depositing extracellular matrix components like fibronectin and collagen, contributing to a non-permissive environment [66].

Interestingly, among previously suggested cell cycle regulation targets was cyclin-dependent kinase 1 (CDK1), which is a key driver of mitosis. CDK1 was suggested to function in coordination with many of our identified hub genes, such as CCNB1, CCNB2, CDC20, and NEK2 [67–69]. Pharmacological inhibitors like olomoucine and flavopiridol were shown to suppress CDK1/2 activity following SCI, reducing glial proliferation and secondary neuronal loss [70, 71]. Roscovitine, another inhibitor, was shown to reduce neuroinflammation and progressive neurodegeneration after traumatic brain injury [72]. Although these inhibitors showed therapeutic potential in preclinical CNS injury models, their lack of specificity for individual CDKs and toxicity-related dosing constraints in clinical trials led to limited clinical translation [73, 74]. This sheds light on the need for more targeted and less toxic therapies to regulate cell cycle dynamics and support regenerative outcomes. Selective inhibition of specific hub genes identified in our study may provide an effective and more refined approach.

To validate the biological roles of the identified hub genes, we suggest that future studies should consider further experimental approaches such as qRT-PCR and immunohistochemistry [1, 75] in order to confirm gene expression patterns. Functional assays like gene knockdown or overexpression in SCI models are also suggested to further assess the impact of hub genes on neuroinflammation, regeneration, and scarring. Similar strategies have been used earlier in previous SCI studies, e.g., [76, 77], and would help confirm the relevance of our findings.

It is worth noting that although activating transcription factor 3 (ATF3) and nidogen 2 (NID2) were identified among the shared oppositely regulated DEG list that was used as input in string for PPI analysis, a closer inspection revealed that the downregulation of these genes was observed only in a single mouse dataset derived exclusively from microglia [27]. ATF3 was consistently upregulated in all other mouse datasets [17, 59] as well as across different species in multiple time points during week 1 post-injury. As for NID2, its downregulation was restricted to the same single mouse dataset derived from microglia-only samples, while being unchanged in any other mouse datasets that recruited all spinal cord tissue for sequencing. This suggests a cell-type-related regulation rather than an overall SCI injury response. Therefore, we recommend cautious interpretation of considering them downregulated in mice during week one after injury.

Among the limitations of our cross-species meta-analysis is the heterogeneity across studies, including variation in injury types (e.g., contusion, transection), injury severity, experimental design, time points (e.g., combined week 1 time points), and evolutionary divergence across species. DEG expression enriched pathways can be influenced by these variations and therefore may affect the comparability and universality of the identified DEG. Although efforts were made to standardize gene selection and enrichment analysis, the biological diversity across different species and models remains a confounding factor that has to be taken in consideration when interpreting the results. Other limitations include sample bias since the majority of the datasets included in this analysis were extracted from rodent SCI models, which, while greatly informative, might not entirely capture the complexity of human responses. Moreover, most selected DEG lists refer to the acute and sub-acute phases after injury, with limited representation of the chronic phase, which could affect the identification of temporally regulated DEG and pathways. Therefore, the translational relevance of findings should be interpreted with caution and validated in human-relevant models. In addition, the limited number of studies (9 studies) in the meta-analysis may limit the universality of the results. Publication bias was not formally evaluated. No contact with the original authors was successfully made to be able to access unavailable DEG lists. Accordingly, this may have resulted in the exclusion of potentially relevant data.

## Conclusion

The results suggest that large gaps still exist in gene expression studies concerning SCI. The limited number of studies highlights the need for more gene expression studies on spinal cord regeneration. Exploiting oppositely regulated genes between spinal cord–regenerating and spinal cord non-regenerating species might open doors for novel perspectives in dealing with SCI.

## Supplementary Information

Below is the link to the electronic supplementary material.

ESM1 (XLSX 72.5 KB)

ESM2 (XLSX 443 KB)

ESM3 (XLSX 132 KB)

ESM4 (XLSX 11.4 KB)

ESM5 (CSV 137 KB)

## Data Availability

No datasets were generated or analyzed during the current study.

## Abbreviations

A. mexicanum: Ambystoma mexicanum
ATF3: Activating transcription factor 3
CCNB1: Cyclin B1
CCNB2: Cyclin B2
CDC20: Cell division cycle 20
CDK1: Cyclin-dependent kinase 1
D. rerio: Danio rerio
DEG: Differentially expressed genes
GEO: Gene expression omnibus
FBXO5: F-box Only protein 5 (also known as EMI1)
KIF23: Kinesin family member 23
MAD2L1: Mitotic arrest deficient-like 1
M. domestica: Monodelphis domestica
M. musculus: Mus musculus
NEK2: NIMA-related kinase 2
NID2: Nidogen 2
Non-REG: Spinal cord non-regenerating
RACGAP1: Rac GTPase activating protein 1
P. marinus: Petromyzon marinus
Pi: Post-injury
R. norvegicus: Rattus norvegicus
REG: Spinal cord regenerating
RRM2: Ribonucleotide reductase regulatory subunit M2
RNA seq: RNA sequencing
SCI: Spinal cord injury
SC RNA seq: Single-cell RNA seq
SN RNA seq: Single-nucleus RNA seq
X. tropicalis: Xenopus tropicalis
